# Doxycycline-Induced Phototoxicity Localized to Vitiligo-Affected and Sun-Exposed Skin

**DOI:** 10.7759/cureus.103789

**Published:** 2026-02-17

**Authors:** Sydney Dong, Claudine Harris, Alexandra Ly, Pamela S Lee

**Affiliations:** 1 Department of Internal Medicine, Harbor University of California Los Angeles Medical Center, Torrance, USA; 2 Department of Infectious Diseases, Harbor University of California Los Angeles Medical Center, Torrance, USA

**Keywords:** doxycycline, drug toxicity, melanin, rash, vitiligo

## Abstract

Vitiligo is a chronic autoimmune disorder characterized by depigmented skin secondary to melanocyte loss, which may increase susceptibility to phototoxic drug reactions. We describe a case of doxycycline-induced phototoxicity selectively affecting vitiliginous and sun-exposed skin, with complete resolution after drug discontinuation. This case highlights melanin’s protective role against ultraviolet-mediated injury and demonstrates that patients with pigmentary disorders may be at higher risk for localized phototoxic reactions from photosensitizing medications. Clinicians should maintain vigilance, emphasize photoprotection, and consider alternative therapies with a lower risk of phototoxicity in such populations.

## Introduction

Vitiligo is a chronic, acquired pigmentary disorder characterized by the selective destruction of epidermal melanocytes, resulting in well-demarcated depigmented macules and patches. Nonsegmental vitiligo, the most common subtype, accounts for approximately 85%-90% of cases and typically appears symmetrically on sun-exposed or friction-prone areas such as the face, hands, and joints [1]. The global prevalence of vitiligo is estimated at 0.5%-2%, with no clear sex or ethnic predilection [2]. Vitiligo carries a significant psychosocial burden due to its visible nature, often leading to social anxiety and reduced quality of life [1,2].

The absence of melanin in vitiliginous patches of skin may increase local vulnerability to phototoxic damage, specifically from ultraviolet (UV) radiation and photosensitizing medications [3]. Phototoxicity occurs when a drug or its metabolites absorb UV radiation, producing reactive oxygen species (ROS) that damage keratinocytes [4]. Normally, melanin absorbs UV light and scavenges ROS, reducing oxidative stress [5]. As a result, vitiliginous skin lacking the photoprotective effects of melanin may be more susceptible to phototoxic injury. However, phototoxic events in vitiligo are rarely reported. Paradoxically, a study of 136 sun-exposed patients with vitiligo and Fitzpatrick skin types 2 and 3 found no increased risk of photoaging in skin histologic specimens [6].

Drug-induced cutaneous reactions are among the most frequent adverse drug events, accounting for 2%-3% of all hospital admissions and affecting up to 5% of hospitalized patients [7]. These reactions range from mild exanthematous eruptions to severe syndromes such as Stevens-Johnson syndrome and toxic epidermal necrolysis. Drug reactions can be immunologic (allergic or autoimmune) or nonimmunologic (directly cytotoxic or phototoxic) [8]. Tetracyclines, such as doxycycline, are common antibiotics and known photosensitizing agents used to treat chronic dermatologic and infectious conditions. Tetracyclines, including doxycycline, may cause cutaneous reactions such as maculopapular and erythematous rashes, as well as phototoxicity. Doxycycline-induced phototoxicity typically presents as erythema, edema, or bullae, specifically in sun-exposed skin [9].

Drug-induced photosensitivity and vitiligo-associated photosensitivity can be distinguished through clinical presentation, distribution patterns, and tools such as the Naranjo adverse drug reaction probability score. Drug-induced photosensitivity causes a sunburn-like reaction that occurs in sun-exposed areas following exposure to both a photosensitizing medication and UV radiation. The eruption typically appears within days of combined drug and light exposure, affects exposed skin in a characteristic distribution (face, neck, dorsal hands, and forearms), and may spare photoprotected areas such as skin covered with clothing. In contrast, vitiligo is characterized by well-demarcated patches that lack melanin protection and are, therefore, more susceptible to sunburn with UV exposure. The Naranjo adverse drug reaction probability score [10] can help establish causality for suspected drug-induced photosensitivity by systematically evaluating factors such as the temporal relationship between drug exposure and reaction onset, improvement after drug withdrawal, recurrence upon rechallenge, and exclusion of alternative causes. Patients with lighter complexions (Fitzpatrick skin types 1 and 2) may be at higher risk of phototoxic reactions with doxycycline [11]; however, selective involvement of depigmented skin has not been previously described.

We present a unique case of doxycycline-induced phototoxicity confined to vitiliginous skin, which highlights the intersection of pigmentary disorders and photosensitizing medications and emphasizes the need for increased awareness in prescribing practices.

## Case presentation

A 51-year-old man with nonsegmental vitiligo, type 2 diabetes, end-stage renal disease on hemodialysis, and a history of methicillin-resistant Staphylococcus aureus bacteremia complicated by L3-L4 discitis and osteomyelitis presented to the Infectious Disease clinic with a three-month history of rash on his hands. Four months prior to presentation, he was transitioned from vancomycin to long-term suppressive doxycycline following hospitalization for progressive discitis and intradiscal abscess. One month after initiating doxycycline, he developed nonpainful, blood-filled blisters that progressively spread on his bilateral hands. He denied similar eruptions in the past. There were no changes in other medications, no reported increase in sun exposure, and no systemic symptoms concerning for infection, malignancy, metabolic disturbance, or autoimmune disease.

**Figure 1:**
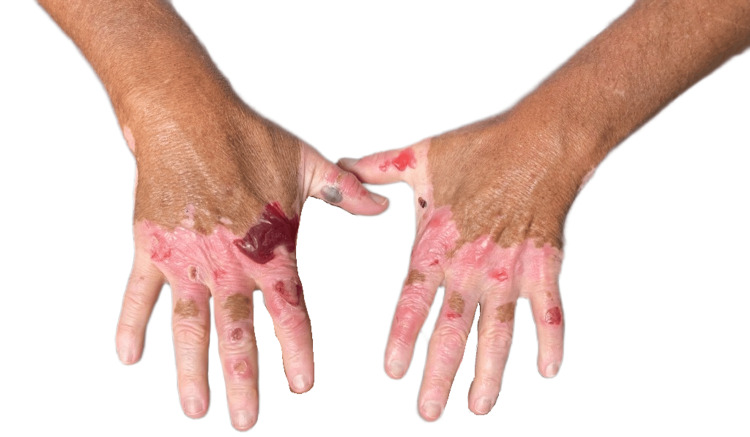
Bilateral dorsal hands with hemorrhagic and erythematous bullae confined to depigmented skin affected by nonsegmental vitiligo

On physical examination, there were scattered intact hemorrhagic bullae, up to 2-3 cm in size, at varying stages of evolution with areas of postbullous desquamation and reepithelialization, localized exclusively to the depigmented skin of his dorsal hands (Figures 1A, 1B). Nikolsky’s sign was negative. The depigmented skin on his feet was spared. There was no necrosis, lymphadenopathy, or mucosal involvement. Laboratory testing, including thyroid function, was otherwise noncontributory (Table 1). Dermatology was consulted, and biopsy was deferred, given high suspicion for doxycycline-induced phototoxicity. Doxycycline was discontinued and replaced with clindamycin, and the patient was advised to avoid further sun exposure while the lesions healed. The lesions completely resolved one month after doxycycline discontinuation.

**Figure 2:**
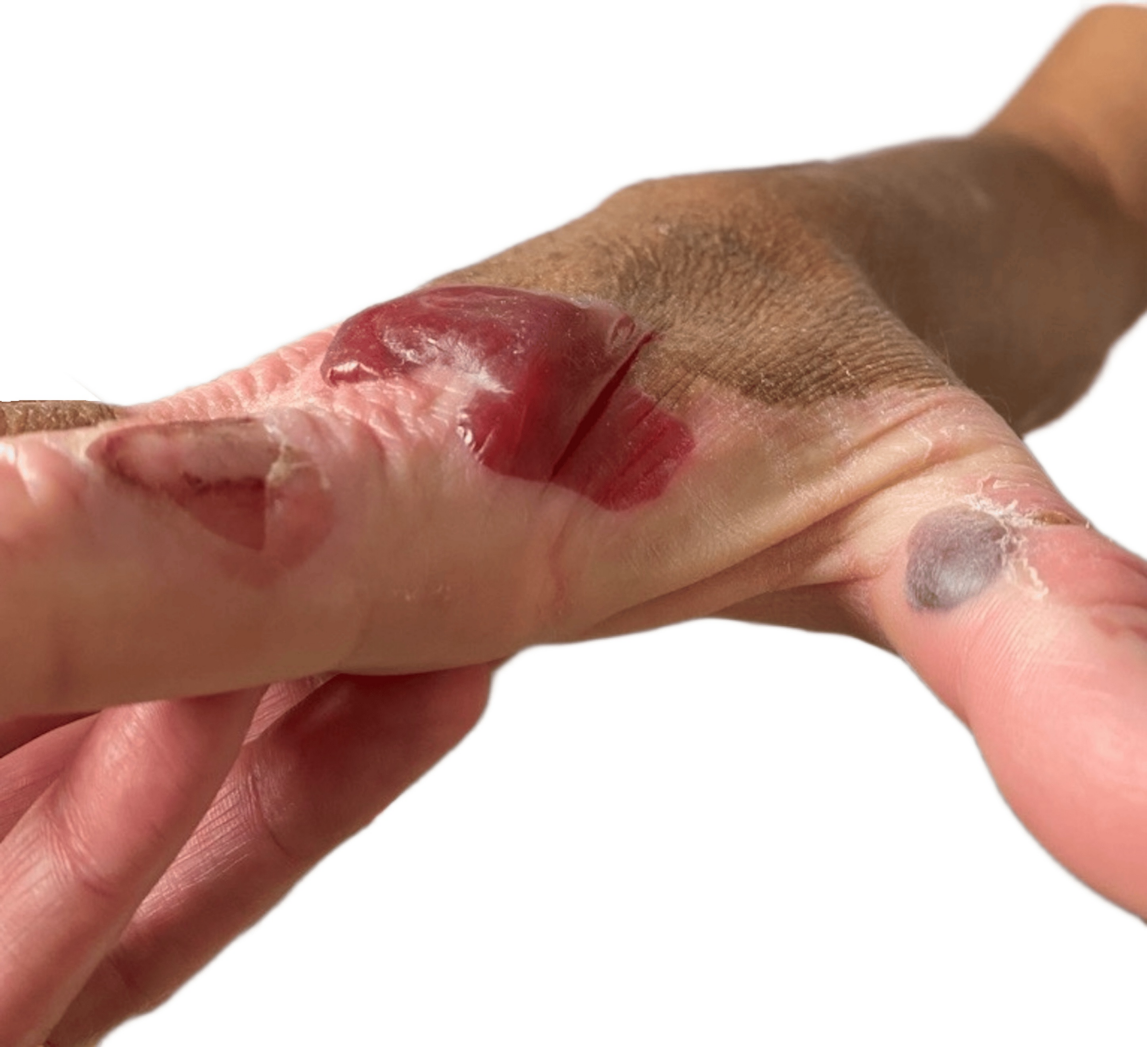
Right dorsal hand with tense, hemorrhagic bullae over the metacarpophalangeal and proximal interphalangeal joints, extending into the Interdigital web spaces of the first and second digits. Lesions are confined to vitiligo-affected skin. Adjacent areas show ruptured bullae with postbullous desquamation and early reepithelialization

**Table 1:** Selected laboratory values at time of rash: laboratory evaluation showed mild leukopenia and anemia with renal function consistent with his baseline in the setting of end-stage renal disease. Liver function and thyroid function were within normal limits. Reference ranges are provided for clinical context

Laboratory test	Patient value	Reference range
White blood cell count	3.0 k/μL	4.0-11.0 k/μL
Hemoglobin	10.6 g/dL	13.0-17.0 g/dL
Platelet count	187 k/μL	150-400 k/μL
Blood urea nitrogen	28 mg/dL	7-20 mg/dL
Creatinine	5.16 mg/dL	0.6-1.2 mg/dL
Glucose	73 mg/dL	70-100 mg/dL (fasting)
Aspartate aminotransferase	16 U/L	10-40 U/L
Alanine aminotransferase	11 U/L	7-56 U/L
Thyroid-stimulating hormone	1.032 μIU/mL	0.4-4.0 μIU/mL

## Discussion

Melanin is crucial for photoprotection, as it absorbs and disperses UV radiation, neutralizes ROS, and limits oxidative DNA damage in epidermal cells. In vitiligo, melanocyte loss removes this natural defense. Doxycycline-induced phototoxicity, including the presence of bullous lesions, is well documented, with reported incidence rates between 3% and 20% depending on dose, treatment duration, and UV exposure [12]. Doxycycline causes dose- and UV-dependent phototoxic reactions wherein, upon UV exposure, doxycycline molecules generate ROS and free radicals that directly damage keratinocytes [4,12].

Localization of doxycycline-related photosensitive rash to vitiliginous skin has not been previously reported. We suspect that our patient’s rash resulted from the initiation of doxycycline and was localized to vitiliginous patches, with sun exposure, due to a lack of melanin production in depigmented skin. Factors supporting our hypothesis include that the patient’s rash started after initiating doxycycline and resolved shortly after stopping it, and that his rash was confined only to sun-exposed areas that lack melanin, sparing both non-sun-exposed areas with vitiligo (e.g., his feet) and sun-exposed areas that have melanin (e.g., nonvitiliginous portions of his hands). Further supporting a link between doxycycline and the patient’s presentation is the known predilection of doxycycline-related rashes to involve the bilateral hands as an early and specific indicator of doxycycline-induced phototoxicity [13].

Management of doxycycline-induced phototoxicity involves discontinuation of the offending agent, symptomatic care with topical corticosteroids and emollients, and strict photoprotection through broad-spectrum sunscreens and protective clothing [8]. Nonphotosensitizing antibiotic alternatives may be considered depending on the indication and tolerance. Patient counseling should emphasize avoiding direct sunlight, even during brief exposures.

Diagnosing drug-induced phototoxicity in the setting of vitiligo requires high clinical suspicion and correlation. Key features of this condition include onset following medication exposure, confinement to sun-exposed areas, and sparing of normally pigmented skin. Histopathology, if performed, typically demonstrates keratinocyte necrosis and vacuolar alteration without eosinophilic infiltration, differentiating it from photoallergic or autoimmune blistering disorders [4]. Recognition may be challenging, as erythema and early inflammation can be subtle in depigmented skin [2]. The development of painful or hemorrhagic lesions on depigmented skin can further exacerbate self-consciousness or emotional distress, known to be prevalent in vitiligo. Supportive counseling and reassurance regarding the reversible nature of the drug reaction are, therefore, integral components of care.

Phototoxic reactions are often underreported or mistaken for sunburn, especially in lighter phototypes or conditions with pigmentary loss [12]. Clinicians should maintain awareness when prescribing long-term photosensitizing drugs, such as tetracyclines and thiazides. These patients may represent an at-risk population due to the absence of melanin and altered oxidative defense mechanisms.

This case demonstrates a previously undescribed intersection between pigmentary disorders and drug-induced phototoxicity, reinforcing the importance of personalized prescribing in dermatology. Future studies could explore whether quantitative differences in melanin density correlate with the severity of phototoxic reactions, providing a mechanistic understanding of pigment-dependent drug toxicity. Improved reporting of pigmentary disorders in drug safety data could help increase recognition and prevention of similar cases.

## Conclusions

This case illustrates an uncommon presentation of doxycycline-induced phototoxicity localized to vitiligo-affected skin, highlighting melanin’s protective role against UV-mediated injury. The absence of pigment likely intensified local ROS-mediated damage, resulting in lesions confined to depigmented areas. Clinicians should exercise caution when prescribing photosensitizing medications to patients with pigmentary disorders, emphasize photoprotection, and ensure close follow-up. Broader awareness and further investigation into pigment-related susceptibility are essential for prevention of drug phototoxicity and safer dermatologic care.
